# Status of national research bioethics committees in the WHO African region

**DOI:** 10.1186/1472-6939-6-10

**Published:** 2005-10-20

**Authors:** Joses M Kirigia, Charles Wambebe, Amido Baba-Moussa

**Affiliations:** 1 World Health Organization, Regional Office for Africa, Brazzaville, Congo; 2 International Biomedical Research in Africa, Abuja, Nigeria

## Abstract

**Background:**

The Regional Committee for Africa of the World Health Organization (WHO) in 2001 expressed concern that some health-related studies undertaken in the Region were not subjected to any form of ethics review. In 2003, the study reported in this paper was conducted to determine which Member country did not have a national research ethics committee (REC) with a view to guiding the WHO Regional Office in developing practical strategies for supporting those countries.

**Methods:**

This is a descriptive study. The questionnaire was prepared and sent by diplomatic pouch to all the 46 Member States in the WHO African Region, through the WHO country representatives, for facilitation and follow up. The data were entered in Excel spreadsheet and subsequently exported to STATA for analysis. A Chi-Squared test (*χ*^2^) for independence was undertaken to test the relationship between presence/absence of Research Ethics Committee (REC) and selected individual socioeconomic and health variables.

**Results:**

The main findings were as follows: the response rate was 61% (28/46); 64% (18/28) confirmed the existence of RECs; 36% (10/28) of the respondent countries did not have a REC (although 80% of them reported that they had in place an ad hoc ethical review mechanism); 85% (22/26) of the countries that responded to this question indicated that ethical approval of research proposals was, in principle, required; and although 59% of the countries that had a REC expected it to meet every month, only 44% of them reported that the REC actually met on a monthly basis. In the Chi-Squared test, only the average population in the group of countries with a REC was statistically different (at 5% level of significance) from that of the group of countries without a REC.

**Conclusion:**

In the current era of globalized biomedical research, good ethics stewardship demands that every country, irrespective of its level of economic development, should have in place a functional research ethics review system in order to protect the dignity, integrity and safety of its citizens who participate in research.

## Background

*"Regrettably, *(over) *50 years after the Nuremberg trials and the Nuremberg code, unethical *(bio)*medical research on humans continues, even in highly privileged countries" *[1]

Biomedical research involves research on pharmaceuticals, medical devices, medical radiation and imaging, surgical procedures, medical records and biomedical samples, as well as epidemiological, social, and psychological investigations [2]. It often entails collection, analysis, and interpretation of information obtained from human beings. Research must be undertaken in an ethical manner so that it assures protection of the dignity, integrity, and safety of all actual or potential research participants [3].

A recent revelation that about 25% of health-related studies in developing countries were not subjected to some form of ethics review by an international review board, national ethics board, or ministry/department of health is worrisome [4]. A similar concern was expressed by the Regional Committee for Africa of the World Health Organization (WHO) in 2001.

Since the end of World War II, ethical and scientific standards for conducting biomedical research on human subjects have been enshrined in international guidelines, including the Nuremberg Code [5], the Declaration of Geneva [6], the International Covenant on Civil and Political Rights [7], the International Code of Medical Ethics of the World Medical Association [8], the Declaration of Helsinki [9], the CIOMS International Ethical Guidelines for Biomedical Research Involving Human Subjects [3], the WHO Operational Guidelines for Ethics Committees that Review Biomedical Research [2] and the ICH Guidelines for Good Clinical Practice [10].

Although there is variance in the scope and emphasis of the above-mentioned international instruments on the ethics of medical research, they all require ethical justification and scientific validity of research; ethical review; informed consent; vulnerability of individuals, groups, communities and populations; women as research subjects; equity regarding burdens and benefits; choice of control in clinical trials; confidentiality; compensation for injury; strengthening of national or local capacity for ethical review; and obligations of sponsors to provide health care [3].

The growing volume of collaborative biomedical studies involving national, multinational and transnational partners developing various interventions targeted against health conditions such as HIV/AIDS, malaria, tuberculosis, childhood illnesses, and causes of maternal morbidity and mortality contained in the Millennium Development Goals, and the potential for exploitation in such research, make it essential for every country to have a functional Research Ethics Committee (REC). The purpose of national REC is to contribute independently, competently, and efficiently to safeguard the dignity, rights, safety, and well-being of all actual or potential research participants; and ensuring the highest attainable quality in the science and ethics of biomedical research [2] in the country.

The aim of the study reported here was to ascertain which Member State in the WHO African Region had a national bioethics committee and which did not (as at 2003), in order to guide the Organization in its support to the establishment of RECs, as well as their strengthening wherever they existed.

## Methods

The questionnaire was deliberately kept short and simple in order to ensure quick response. It consisted of questions aimed at obtaining the following information: existence of a national ethics committee dealing with health issues; its composition and main functions; frequency of scheduled meetings and number of times the committee actually met last year; if a REC did not exist, were there mechanisms for clearing ethical issues in research; and whether a national ethical approval was required for implementation of research proposals.

The questionnaire was developed in French and subsequently translated into English and Portuguese. Of the 46 Member countries in the WHO African Region, 21 speak French, 20 English and 5 Portuguese. It was sent by WHO diplomatic pouch to each of the 46 countries through the WHO country representatives for facilitation and follow up. The data were entered in Excel spreadsheet and subsequently exported to STATA for analysis.

A Chi-Squared test (*χ*^2^) for independence was undertaken to test the relationship between presence/absence of Research Ethics Committee (REC) and selected individual socioeconomic and health variables. The null hypothesis (H_0_) is that there is no difference between the mean of a given socioeconomic or health variable among the group of countries with REC and the group without REC. The alternative hypothesis (H_A_) is that there is difference between the mean of a given socioeconomic or health variable among the group of countries with REC and the group without REC. Thus, for maternal mortality per 100000 live births, the hypotheses are as follows: (i) H_0_: There is no difference in average maternal mortality ratio between the group of countries with REC and those without; and (ii) H_A_: There is difference in average maternal mortality ratio between the group of countries with REC and those without. If the computed Chi-square () is greater than critical Chi-square (), then we reject the null hypothesis and accept the alternative hypothesis at a given level of significance, e.g. 95% confidence level or 5% level of significance. On the other hand, if the computed Chi-square () is less than critical Chi-square (), then we accept the null hypothesis, i.e. there is no reasonable evidence to reject the H_0_.

The data on maternal mortality per 100000 live births, population in a country, life expectancy at birth, probability of dying (per 1000) below age 5 years, probability of dying (per 1000) between age 15–60 years and infant mortality per 1000 live births were obtained from the World Health Report 2005 [19]. While the data on gross national income per capita (US$), percentage of population aged 15 years and above that is illiterate, and gross primary enrolment (% of school age population) were taken from the World Bank website [20].

## Results

A total of 28 (60.9%) of the 46 countries completed the questionnaire and returned it to the investigators. Response rates of 50% (10/20), 67% (14/21) and 80% (4/5) were recorded for the English, French and Portuguese speaking countries respectively. An average of three remainders were sent through WHO Country Representatives to non-responding countries.

Of the 28 countries that responded, 64% (18/28) confirmed the existence of a national REC dealing with bioethics research issues (Table 1). This meant that 36% (10/28) of the respondent countries did not have an ethics committee. Sixty-seven per cent (12/18) of the countries that did have an ethics committee had it named as national bioethics committee; the remaining 33% (6/18) had alternative names for their national entity that performed the functions of REC.

**Table 1:** Presence/absence of RECs

**Country**	**Presence of REC**
Algeria	Yes
Angola	Yes
Botswana	Yes
Burkina Faso	Yes
Cameroon	Yes
Cape Verde	No
Chad	No
Congo	No
Democratic Republic of Congo	Yes
Ethiopia	Yes
Gambia	Yes
Guinea	Yes
Guinea Bissau	No
Guinea Equatorial	No
Kenya	Yes
Malawi	No
Mali	Yes
Mauritania	No
Mauritius	Yes
Mozambique	Yes
Niger	Yes
Rwanda	Yes
Sao Tome et Principe	No
Seychelles	Yes
Swaziland	No
Togo	No
Zambia	Yes
Zimbabwe	Yes

Source: Survey data

As indicated in Table 2, the reported composition of REC membership varied widely. Over 50% of the countries had specialists from national medical research council/institute, universities and social sciences, and public health professionals from ministry of health.

**Table 2:** Composition of research ethics committees in countries that reported their existence

**Cadre**	**Percentage (Frequency)**
National medical research institute/council	72% (13/18)
University	61% (11/18)
National medical association	44% (8/18)
Renowned researcher	33% (6/18)
Social scientist	50% (9/18)
Public health professional from ministry of health	89% (16/18)
National bureau of standards	11% (2/18)
WHO	5% (1/18)
Attorney-general	39% (7/18)
Others	83% (15/18)

The number of members on the national committee ranged from 4 to 37. The average membership was 11, with a standard deviation of 8.

The countries that confirmed the existence of a REC were requested to name its main functions. Their responses are summarized in Table 3. All these countries (18/18) indicated 'Review and approve all research protocols on human subjects' as one of the main functions of REC.

**Table 3:** Main functions of research ethics committees in countries that reported their existence

**Functions**	**Percentage (Frequency)**
Review and approve all research protocols on human subjects	100% (18/18)
Ensure that projects sponsored by external donors are submitted for approval to ethical clearance committee of the initiating country	5% (1/18)
Build the capacity of institutional and regional ethical clearance committees	11% (2/18)
Give clearance for export of samples or specimens for research related to human health	5% (1/18)
Coordinating and promoting research	22% (4/18)

Eighty per cent (8/10) of the countries that did not have a REC reported that they had ad hoc mechanisms for ethical review of bioethics research protocols. These consisted of mainly hand-picking a few colleagues at the ministry of health to review proposals whenever they were submitted.

The countries were asked whether REC was supposed to meet monthly, quarterly, twice a year, once a year, or on demand. Seventeen countries responded to this question; of them, ten indicated the frequency as monthly, three quarterly, one twice a year, and three on demand.

The countries were also requested to indicate the actual frequency of REC meetings during the last year. A total of 16 countries responded to this question; of them, seven reported as monthly, five quarterly, three twice a year, zero annually, and one indicated that their REC had never met.

Lastly, all countries were asked whether ethical approval for research proposals was actually required. Eighty-five per cent (22/26) of the countries that responded to this question indicated that an approval was required.

Table 4 presents the Kruskal-Wallis test of equality of populations means of socio-economic and health variables. This test was undertaken to test the relationship between presence/absence of REC and the individual socio-economic and variables. The p value reported in the third column of Table 4 is the estimated probability of rejecting the null hypothesis (H_0_) when the hypothesis is true. The computed Chi-squared value of the population variable was greater than the critical Chi-squared value; thus we can conclude that the average population in the group of countries with a REC was statistically different from that of the group of countries without a REC. On the other hand, since the computed values for all the other socio-economic and health variables were less than their respective critical Chi-squared values (at 5% level of significance) we accept the H_0_.

**Table 4:** Kruskal-Wallis pairwise comparisons between variables of the group of countries with a research ethics committee and the group without

**Variables**	**Chi-squared statistic**	**p-value**	**Decision at 5% significance level**
Maternal mortality per 100000 live births	0.026144	P = 0.8712	not significant, accept H_0_
Population in a country	7.468966	P = 0.0063	Significant, reject H_0_
Life expectancy at birth	0.7468966	P = 0.3876	not significant, accept H_0_
Probability of dying (per 1000) below age 5 years	0.331125	P = 0.565	not significant, accept H_0_
Probability of dying (per 1000) between age 15–60 years	0.186207	P = 0.6661	not significant, accept H_0_
Gross national income per capita (US$)	0.111875	P = 0.738	not significant, accept H_0_
Infant mortality per 1000 live births	0.028161	P = 0.8667	not significant, accept H_0_
Percentage of population aged 15+ years that is illiterate	0.22623	P = 0.6343	not significant, accept H_0_
Gross primary enrolment (% of school age population)	2.712306	P = 0.0996	not significant, accept H_0_

Note: Total number of observations = 28.

## Discussion

### Key findings

The purpose of this study was to ascertain which country in the WHO African Region had a national bioethics committee and which did not. The key findings were as follows: (i) the response rate was 61% (28/46); (ii) 64% (18/28) confirmed the existence of a national ethics committee; (iii) 36% (10/28) of the respondent countries did not have an ethics committee (although 80% of them reported that they used an ad hoc ethical review mechanism); (iv) 85% (22/26) of the countries that responded to this question indicated that ethical approval of research proposals was, in principle, required; and (v) although 59% of the countries expected their REC to meet every month, only 44% reported that it actually met on a monthly basis.

### Implications

#### Response rate

It is very sad that only 28 out of 46 countries responded to the questionnaire. It would have been preferable to have responses from all the 46 countries. This would have provided WHO and the Special Programme for Research and Training in Tropical Diseases Research (TDR) with a strong basis for planning support to the countries in the African Region that needed it to develop or strengthen their ethical review systems.

Thus, the analysis reported in this paper was confined to the 28 countries that responded and mainly to the 18 who reported to have had a REC. We are not certain why 18 countries did not respond to the questionnaire. However, it could partly be attributed to lack of a culture for generating and utilizing evidence in decision-making in the Region.

#### Existence of REC

Although from this study we cannot assess the functional status of RECs in the 18 countries that reported their existence, we are more concerned about the remaining ten countries that did not have a national ethics committee in an era in which biomedical research (and science in general) has become increasingly globalized. This is quite disconcerting in a Region where 39% of the adult population is illiterate and 44% live below the international poverty line of US$1 per day [11]. They thus are largely unaware of their basic human rights, including the right to refuse to participate in health research. In the absence of any checks and balances due to lack of functional RECs, the populations in these countries run the risk of being abused by unscrupulous researchers [1,2,12].

WHO is currently supporting Member countries without RECs to establish them and those with RECs to improve their performance. Given the critical importance of RECs, we do hope that the ongoing support to countries in the African Region would be accelerated and sustained.

#### Functions/roles of REC

The roles of ethical review committees are to: ensure that all proposed interventions (drugs, vaccines, medical devices or procedures) are safe; ensure that proposed research is scientifically sound; ensure that all other ethical concerns arising from a protocol are satisfactorily resolved both in principle and in practice; and ensure competence of investigators; keep records of decisions; monitor and audit the conduct of ongoing research projects [3]; "... evaluate research proposals with special attention to risk/benefit ratios, equity in distribution of benefits and burdens, potential conflicts of interests, the adequacy of information provided for subjects, and the protection of freedom: within the consent process; enable study subjects to withdraw without prejudice to care; and persuade investigators to publish, to educate and assist faculty, researchers and community in understanding and appreciating the ethics of research" [1].

It was beyond the scope of the current study to assess the extent to which RECs in the Region performed the above-mentioned roles. However, it appeared that all the respondent countries regarded the main function of REC to be to review and approve research protocols on human subjects. There was no mention during the survey that RECs were expected to also monitor the actual conduct of research to ensure justice in the distribution of costs and benefits. We concur with Benatar [1] that "lack of attention to how research is actually being conducted is a serious shortcoming, requiring critical attention in an era of expanding research, growing links with industry and commercial organizations, documented inadequacies in the protection of research subjects and with growing recognition of the need to avoid exploitation." In short, it is vitally important for RECs to ensure that they competently implement all the guidelines stipulated by the Council for International Organizations of Medical Sciences (CIOMS) [3].

#### Composition of REC

The CIOMS guidelines [3] recommend that the membership of REC "... should include physicians, scientists and other professionals such as nurses, lawyers, ethicists and clergy as well as lay persons qualified to represent the cultural and moral values of the community." The survey undertaken in the current study did not reveal the involvement of nurses, ethicists and qualified laypersons in the work of RECs. Since nurses constitute the largest group of health personnel in health systems delivery and play a critical role as members of the health team, it is vitally important that they are involved in all ethical review processes. Also, non-inclusion of qualified laypersons in RECs may compromise cultural and moral values [1].

#### Ethical approval

About 15% of the respondent countries indicated that ethical approval of research proposals was not required. In such countries, the health and safety of persons participating in research was not assured. Even among the countries that indicated that ethical approval of research proposals was, in principle, required, we were not able to determine: (i) what proportion of research protocols were actually approved before implementation; and (ii) what proportion of the approved studies were actually monitored by REC throughout the research project cycle, i.e. protocol design, data collection and archival, data analysis, dissemination of results, etc.

#### Frequency of REC meetings

In the current study, the frequency of REC meetings was used both as an indicator of their functionality and as an indirect proxy of performance. Half of the countries that responded to this issue reported that their RECs met either quarterly (31%) or twice a year (19%). In our view, the 3 to 6-month interval for REC meetings was too long as this may result in unnecessary delays in processing (approval or rejection) of research protocols. Where the sponsors of research projects have stringent deadlines, such delays may: (i) lead to withdrawal of research funding; (ii) reduce the probability of researchers from concerned countries getting funding in the future; (iii) increase the cost of research; (iv) hamper the development and availability of public health interventions; and (v) provide adverse incentives for some researchers to undertake research covertly, without ethical review, especially in settings where appropriate legislations either do not exist or are not policed.

One of the countries indicated that its REC had never met. On hindsight, we feel we should have included questions to probe the reasons for not holding REC meetings. At the moment, it is not possible to know whether the absence of meetings was due to lack of protocols to review, lack of resources to organize the meetings, lack of quorum, incompetent leadership, or due to some other logistical reasons.

### Agenda for action

The following actions need to be taken by countries in the African Region:

• Countries should adapt appropriately international guidelines for biomedical research involving human subjects and make them available to all national health and health-related research institutions and health facilities.

• As a good national ethics steward, every country should ensure that it has an operational bioethics research review system in place, which includes national, regional, district and institutional (health facility) ethics committees.

• All countries should make sure that there are appropriate policies and legislations to guide and reinforce national bioethics research review systems.

• All countries should ensure that the composition and mandates of RECs concur with international standards, while providing institutional and financial resources to ensure independent and competent execution of all their roles. In this regard, we agree with Benatar [1] that all countries should 'develop the expertise and infrastructure required to (i) evaluate ethical problems; and (ii) educate practitioners and researchers and facilitate development of policy'.

• Each country should champion 'institutionalization of training in ethics and human rights in relation to health at all stages of the education and training of all health workers, including medical, public health and nursing schools' [13].

• All countries with RECs should develop mechanisms for monitoring and auditing their work to ensure that they are guaranteeing research adherence to all the CIOMS guidelines [3].

### Further research

Our suggestions for further research in countries in the African Region are as follows:

#### Situation analysis

A detailed survey and evaluation should be undertaken of the effectiveness and efficiency of the entire national ethics review system (including regional, district, institutional and community-based RECs) in implementing the CIOMS guidelines [3]. The evaluation can be accomplished using the WHO Guidelines [14]. It would be important to evaluate: (i) the organization, financing and functionality of the whole ethics review system; (ii) the extent to which RECs are involved in monitoring every stage/step in a research project cycle, including protocol design, implementation, archival of data, analysis and public dissemination of results; (iii) the effects of recent information and technology developments on RECs' *modus operandi*; and (iv) challenges faced by RECs.

#### Best practices

A detailed study and documentation of best-performing research ethical review systems (including RECs) in the Region should be conducted with a view to drawing lessons that other countries can emulate.

#### Principal-agency relationship

The principal-agency relationship between ethics committees and human research subjects should be established.

#### Institutionalisation of ethics education and training

A review of the existing international ethics review guidelines should be undertaken with a view to designing appropriate undergraduate and postgraduate curricula on research ethics. We concur with Fischer and Zigmund [15] that research ethics should be taught throughout the graduate curriculum. However, in addition, we are of the opinion that in Africa where a majority of the health and allied sciences undergraduates do not proceed to postgraduate studies, it is critically important to introduce undergraduates also to research ethics. After obtaining their degrees, most undergraduates are normally deployed in rural areas where, by virtue of being the most educated, they often bear the burden of assuring that human rights of their actual and potential clients are respected and protected in the course of their clinical work and research carried out by others.

#### Ways of improving REC performance

There is need for studies that explore the cost and benefits (effectiveness) of alternative ways of leveraging the recent advances in technology (teleconferencing, video conferencing, e-mail) to boost the work of RECs. Where these technologies exist, they would not only reduce the cost of face-to-face meetings but will also ensure timely review of research protocols.

#### Partnerships

An exploration of the modalities of South-South and North-South cooperation to strengthen the capacities of bioethics review systems in the Region should be made. For example, the WHO Regional Committee for Africa [16] identified the need for effective inter-country mechanisms to monitor health research in order to ensure that existing national and international bioethics guidelines were adhered to. In addition, countries with limited bioethics capacities could easily tap into the internationally available bioethics expertise through the Internet. We are encouraged by the emphasis laid on capacity-building for national ethics committees by the European and Developing Countries Clinical Trials Partnership (EDCTP).

#### Financing of REC work

Currently, the effectiveness of RECs in many countries is greatly constrained by lack of resources [17]. Thus, there is urgent need for research into finding innovative mechanisms for ethically financing REC activities.

## Conclusion

In the current era of globalized research, good ethics stewardship demands that every country, whatever its level of economic development, should have a functional research ethics review system for protecting the dignity, integrity and health safety of all its citizens participating in research. Those countries that do not currently have such systems should urgently leverage the services provided by the WHO Strategic Initiative for Developing Capacity in Ethical Review (SIDCER) [18] to develop capacities for ensuring ethical research practices.

## Competing interests

The author(s) declare that they have no competing interests.

## Authors' contributions

JMK recoded the raw data, did the analysis and participated in drafting of all sections of the document. CW participated in the analysis and drafting of all sections of the document. AB sent out the questionnaires and made follow-up through WRs. He also participated in drafting the Background and Methods sections. The WHO Country Representatives of the 28 countries that responded ensured that the relevant persons in their countries of duty completed the questionnaires. All authors read and approved the final manuscript.

## Pre-publication history

The pre-publication history for this paper can be accessed here:



## References

[B1] Benatar SR (2002). Reflections and recommendations on research ethics in developing countries. Social Science and Medicine.

[B2] World Health Organization (2000). Operational guidelines for ethics committees that review biomedical research Geneva.

[B3] CIOMS (2002). International ethical guidelines for biomedical research involving human subjects Geneva.

[B4] Hyder AA, Wali SA, Khan AN, Teoh NB, Kass NE, Dawson L (2004). Ethical review of health research: a perspective from developing country researchers. Journal of Medical Ethics.

[B5] United States Government Printing Office (1949). Trials of War Criminals before the Nuremberg Military Tribunals under Control Council Law No 10, Washington, DC.

[B6] World Medical Association (1948). Declaration of Geneva – physician's oath Geneva.

[B7] United Nations (1966). International covenant on civil and political rights New York.

[B8] World Medical Association (1949). International code of medical ethics of the World Medical Association. World Medical Association Bulletin.

[B9] World Medical Organization (1996). Declaration of Helsinki. British Medical Journal.

[B10] International Conference on Harmonization (ICH) (2002). Guidelines for good clinical practice Toronto.

[B11] United Nations Development Programme (2003). Human Development Report 2003: Millennium Development Goals: a compact among nations to end human poverty New York.

[B12] Weindling P (1996). Human guinea pigs and the ethics of experimentation: the BMJ's correspondent at the Nuremberg medical trail. British Medical Journal.

[B13] CIOMS (1997). CIOMS-WHO Initiative on ethics, equity and health for all Geneva.

[B14] World Health Organization (2002). Surveying and evaluating ethical review practices Geneva.

[B15] Fischer BA, Zigmund MJ (1996). Teaching ethics: resources for researchers. TINS.

[B16] WHO/AFRO (2001). Fifty-first session of the WHO Regional Committee for Africa: final report Brazzaville.

[B17] Dickens BM, Cook RJ (2003). Challenges of ethical research in resource-poor settings. International Journal of Gynaecology and Obstetrics.

[B18] World Health Organization (2002). Strategic Initiative for Developing Capacity in Ethical Review (SIDCER): terms of reference and strategic plan Geneva.

[B19] World Health Organization (2005). World Health Report 2005: making every mother and child count Geneva.

[B20] World Bank http://www.worldbank.org/.

